# Case report: an unusual unilateral pterygium — a secondary pterygium caused by parasitosis in the scleral fistula

**DOI:** 10.1186/s12886-021-02083-2

**Published:** 2021-09-06

**Authors:** Wenjie Zeng, Zhaoyi Pan, Jun Wang, Xianghui Deng, Wenmin Jiang

**Affiliations:** 1 Department of Ophthalmology, The Second Xiangya Hospital, Central South University, Changsha, People’s Republic of China; 2 Hunan Clinical Research Center of Ophthalmic Disease, Changsha, People’s Republic of China

**Keywords:** Scleral fistula, Ocular parasitosis, Secondary pterygium, Unilateral pterygium

## Abstract

**Background:**

Ocular parasitosis can cause eye damage, which contribute to eye symptoms such as burning, itching and even blindness. It is uncommon to see the parasitosis lying in the sclera layer, neither it causing pterygium. Here, we present an unusual case of a secondary pterygium caused by intrascleral worm.

**Case presentation:**

A 52-year-old women complained about discomfort in right eye for 6 years. Slit-lamp examination indicated a thickened triangular layers of conjunctiva extending from the nasal edge to the cornea. The diagnosis was pterygium in the right eye. To our surprise, after scleral of nasal side exposed, we could see a tiny fistula right in the sclera which lied right under the pterygium, with an alive and motile worm inside. An intrascleral fistula was noted. Then the worm was removed by forceps from the fistula, which was creamy white, thread-like and 1 cm long.

**Discussion and conclusions:**

As far as we known, it is the first case of an intrascleral worm hidden beneath the conjunctiva which caused the secondary pterygium. It is hard to know the etiology of the secondary pterygium which caused by parasitosis in the scleral fistula untill excision surgery. It is hard to imagine the worm was living in the sclera of the patient for a long-time.

## Background

Most ocular parasites infections can result from parasitic migration through bloodstream or adjacent tissues in the host, fewer from inoculation in the eye [1]. It can cause eye damage by disrupting normal structure mechanically, secreting toxic metabolites and inducing immune or allergic reactions [2, 3]. Ocular parasites—involving three species: worms, protozoa, and arthropods—are an alive organism living in the host eye that acquire some of its nutritional requirements through intimate contact with the host [4]. Common routes of worm infection include ingesting unclean water, soil, undercooked food and contacting animals that carrying worm eggs [1, 5]. Due to the blood-eye barrier and immunosuppressive micro-environment, worms can remain in the eye for a long time without being killed by ocular immune system.

Pterygium—a conjunctival fibrovascular degeneration disease—may be caused by ultraviolet, increasing age, male sex, outdoor occupation, alcohol use, heredity et al. [6, 7]. It is a chronic inflammation process [8]. Long-term parasites infection may relate to chronic inflammation [9], Studies have suggested that mites may play a role in the development of pterygium [10, 11]. The following is a report of a rare case of intrascleral worm hidden beneath a fleshy pterygium for a time till pterygium excision surgery.

## Case presentation

**Fig. 1:**
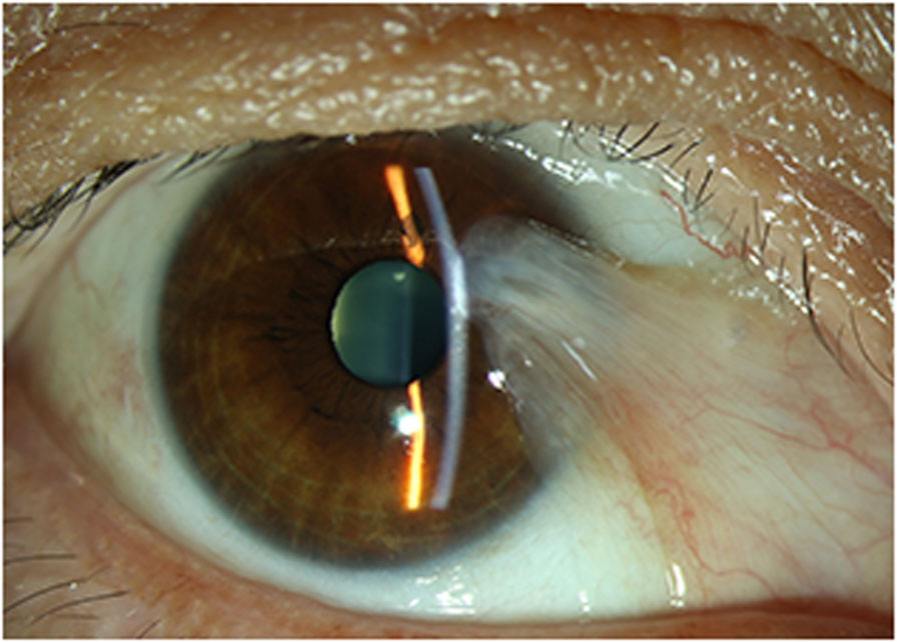
Preoperative image of the patient’s right eye. Slit-lamp examination showing a fleshy nasal pterygium in right eye, extending at 3 o’clock and 2 mm away from the limbus, covering part of corneal pupillary area

A 52-year-old women was referred to our ophthalmology department with discomfort in right eye for 6 years and no other symptoms. Her best-corrected visual acuity was 20/20 OD (+ 1.0 Diopters Sphere) and 20/20 OS (− 0.5 Diopters Sphere), with intraocular pressure of 17 and 18 mmHg respectively. In slit-lamp examination, the patient’s right eye showed a fleshy nasal triangular membrane (Fig. 1), extending at 3 o’clock and 2 mm away from the limbus, covering part of corneal pupillary area. The anterior chamber depth was normal in both eyes. In blood routine, eosinophils and basophils were increased; the ratio of each were 8.50 and 1.50%. After the above examinations, we diagnosed the patient with pterygium in the right eye and decided to undergo excision surgery next day.

**Fig. 2:**
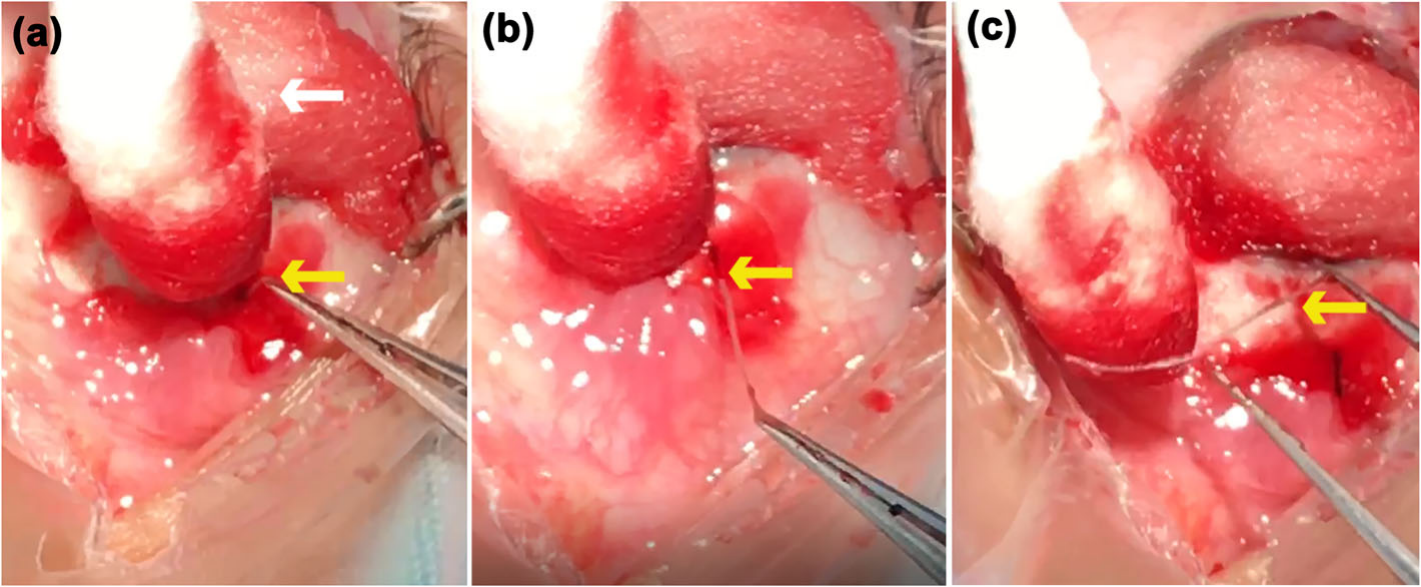
The worm exposing process during the surgery. The patient’s cornea was covered with the cotton ball (**white arrow**); the nasal raised spot (**yellow arrow**) was exposed 3 mm away from the limbus after excising the pterygium (**a**). The alive and motile worm (**yellow arrow**) was being pulled out from the fistula inside the sclera by toothed forceps (**b**). The worm (**yellow arrow**) was completely pulled out from the fistula with a little bleeding, which was creamy white, thread-like, 1 cm long (**c**)

**Fig. 3:**
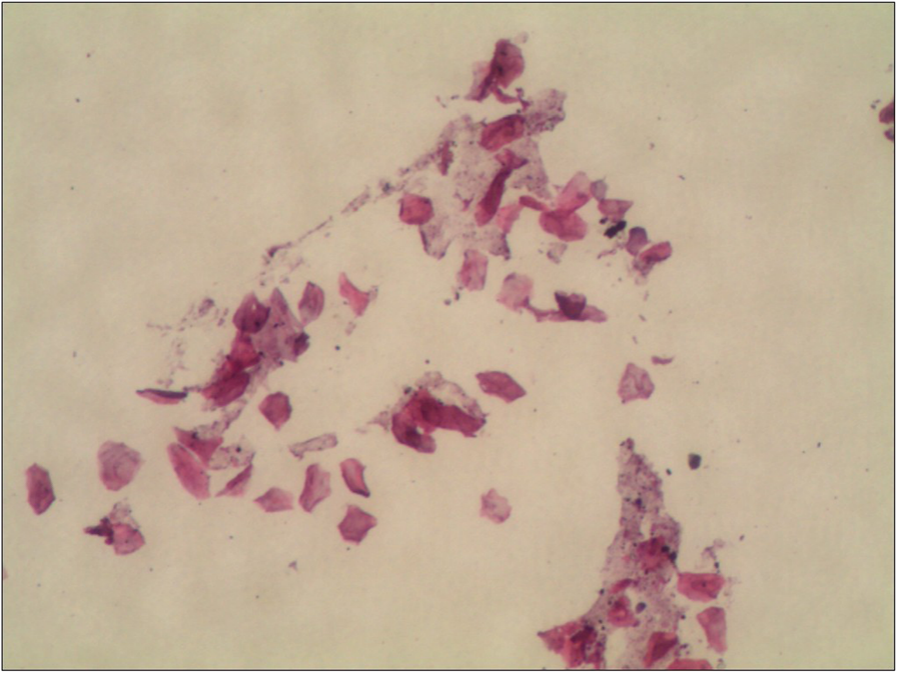
Pathologic finding. Pathological examination of the excised pterygium and the worm showing a few dissociative keratins under the microscope, which may be excluded from the worm

**Fig. 4:**
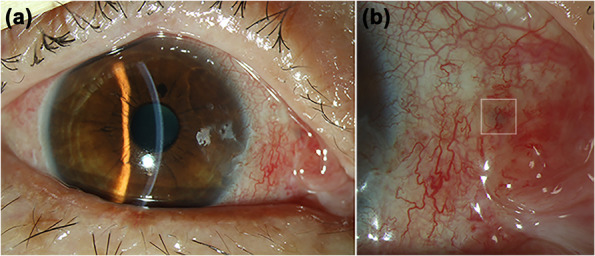
Postoperative image of the patient’s right eye. The incision recovered well after excising the pterygium and removing the worm (**a**). Magnified image shows that the fistula, where the worm living inside, turned into a black spot (**white square**) during the healing process (**b**)

After surgical excision of the pterygium, with the scleral of nasal side exposed, we could see a tiny fistula right in the sclera which lied right under the pterygium (Fig. 2a). We used a toothed forceps to clip the raised spot; creepily an alive and motile worm was pulled out from the fistula with a little bleeding (Fig. 2b). After clipping out the worm, we examined the bare sclera carefully, we found that the fistula was in one of the layer of the sclera with the worm living in. The worm (Fig. 2c) was creamy white, thread-like, 1 cm long, and round with bilateral symmetry. Then we underwent the corneal limbal stem cell autograft for patient to prevent the pterygium from recurring. After surgery, we send the excised pterygium and the worm for pathological examination at our hospital. Unfortunately, the report only showed a few dissociative keratins under the microscope, which may be excluded from the worm (Fig. 3). Postoperatively, the patient received sodium hyaluronate eye drops 4 times per day, deproteinized calfblood extract eye gel 1 times per day, levofloxacin eye drops 3 times per day for right eye about 1 week. 10 days after the operation, the patient re-examined at our ophthalmology clinic, which showed the surgical incision recovering well (Fig. 4a and b).

## Discussion and conclusions

Cases of intrascleral worms have been rarely reported, especially those hidden beneath the pterygium. Most ocular worms can infect the conjunctiva, eyelid, anterior chamber, orbit and retina [12], but rarely sclera, which may due to its dense and crisscrossed fibrosis, preventing most worms from penetrating through the ocular surface.

The origin of the parasitosis was hard to know, we could only surmise it comes from conjunctival vessels, migrating to the surface of the scleral, and caused a fistula. Pterygium is a chronic inflammation process [8], and long-term mite infection can cause ocular chronic inflammation, which may play a role in the development of pterygium. The pterygium recurrence rate was higher in patients with mite infection, which may relate to chronic inflammation mediated by T helper cell 1 7[11]. The secretions and metabolites of demodex may produce more inflammatory factors and vascular endothelial growth factor in the cornea, which stimulate the surrounding conjunctival fibrous tissue and blood vessels to proliferate, thus promoting pterygium course [10].

To the best of our knowledge, this is the first case of an intrascleral worm hidden beneath a secondary pterygium till excision surgery. It also gives us a hint that monocular pterygium maybe secondary caused by parasite infection.

## Data Availability

All data generated or analyzed during this study are included in this published article.
